# Expression profiling of serum inducible genes identifies a subset of SRF target genes that are MKL dependent

**DOI:** 10.1186/1471-2199-5-13

**Published:** 2004-08-25

**Authors:** Ahalya Selvaraj, Ron Prywes

**Affiliations:** 1 Department of Biological Sciences, Columbia University, New York, New York, USA

## Abstract

**Background:**

Serum Response Factor (SRF) is a transcription factor that is required for the expression of many genes including immediate early genes, cytoskeletal genes, and muscle-specific genes. SRF is activated in response to extra-cellular signals by its association with a diverse set of co-activators in different cell types. In the case of the ubiquitously expressed immediate early genes, the two sets of SRF binding proteins that regulate its activity are the TCF family of proteins that include Elk1, SAP1 and SAP2 and the myocardin-related MKL family of proteins that include MKL1 and MKL2 (also known as MAL, MRTF-A and -B and BSAC). In response to serum or growth factors these two classes of co-activators are activated by different upstream signal transduction pathways. However, it is not clear how they differentially activate SRF target genes.

**Results:**

In order to identify the serum-inducible SRF target genes that are specifically dependent on the MKL pathway, we have performed microarray experiments using a cell line that expresses dominant negative MKL1. This approach was used to identify SRF target genes whose activation is MKL-dependent. Twenty-eight of 150 serum-inducible genes were found to be MKL-dependent. The promoters of the serum-inducible genes were analyzed for SRF binding sites and other common regulatory elements. Putative SRF binding sites were found at a higher rate than in a mouse promoter database but were only identified in 12% of the serum-inducible promoters analyzed. Additional partial matches to the consensus SRF binding site were found at a higher than expected rate in the MKL-dependent gene promoters. The analysis for other common regulatory elements is discussed.

**Conclusions:**

These results suggest that a subset of immediate early and SRF target genes are activated by the Rho-MKL pathway. MKL may also contribute to the induction of other SRF target genes however its role is not essential, possibly due to other activation mechanisms such as MAPK phosphorylation of TCFs.

## Background

Quiescent cells exposed to growth factors respond by expressing a variety of immediate early genes (IEG) that do not need new protein synthesis for their expression [1]. Serum or growth factor induced expression of many of these immediate early genes, such as c-fos, egr1, cyr61 and pip92, is dependent on a sequence element in their promoter termed the Serum Response Element (SRE). This sequence element contains an A/T rich core flanked by an inverted repeat and is also known as the CArG box (CC(A/T)_6_GG). The CArG box is specifically bound by Serum Response Factor (SRF) [2-4]. Both the SRE and SRF are required for the serum inducibility of these genes since microinjection of SRE oligonucleotides or anti-SRF antibodies blocked induction in NIH3T3 cells [5]. In addition, mutation of the SRE blocked serum induction of reporter genes containing immediate early gene promoters and SRF null ES cells were defective for immediate early gene activation [6,7].

Although the immediate early genes are so named because of their rapid inducibility after growth factor treatment, different kinetics of expression have been observed among the immediate early genes. Expression of the proto-oncogene c-fos peaks at around 30 minutes after stimulation whereas the peak expression of SRF mRNA occurs after 90–120 minutes [8,9]. Thus SRF has been characterized as a "delayed" IEG although its expression is still independent of new protein synthesis.

Activation of SRF by growth factors occurs through at least two mechanisms – the TCF and RhoA pathways [10,11]. Serum or growth factor induction leads to the phosphorylation of p62TCF by MAP kinases. TCF is a ternary complex factor that binds to both SRF and flanking sequences of the SRE. TCF binding to the SRE requires the prior binding of SRF as well as an adjacent TCF binding site. TCF is encoded by three ets-related genes, Elk1, SAP1 and SAP2/Net [12].

An additional pathway that activates SRF is through activation of the small GTPase RhoA [11]. Activated RhoA induces the expression of SRE reporter genes while inhibition of RhoA blocks serum induction. RhoA also causes the formation of stress fibers and the use of actin filament inhibitors and actin mutants suggests that actin treadmilling can control SRE activation [13,14]. The RhoA effectors mDia and ROCK appear to be involved in regulating both actin treadmilling and SRF activation [15,16]. This has led to a model whereby free G-actin inhibits SRF activation and this inhibition is relieved when G-actin levels are depleted by their polymerization into actin filaments. However, mutants of RhoA have been identified that are defective for SRF activation but still cause the formation of stress fibers and vice versa, suggesting that a pathway exists for RhoA activation of SRF independent of stress fiber formation [17,18].

The use of pathway specific inhibitors has suggested the presence of two types of SRF target genes – those that are largely dependent on the Rho-actin pathway and those that are largely dependent on the MAP kinase pathway [19]. Use of reporter genes suggests that some promoters may be activated by both pathways, but that activation by the Rho pathway is only apparent after mutation of the TCF pathway [20].

We and others have recently identified a family of SRF-specific transcriptional co-activators – the MKL family, comprised of MKL1 and MKL2, that function downstream of the RhoA pathway [21-26]. MKL1 and 2 are widely expressed genes related to the heart and smooth muscle-specific SRF co-activator myocardin [21,22,24-29], thus making them candidate proteins in the signal transduction pathway of immediate early genes. Indeed, experiments using dominant negative proteins and RNA interference have shown that they are required for the induction of the immediate early genes srf, vinculin and c-fos [21,23]. Serum induction of SRF and vinculin was dependent on MKL1 both at the early and late time points of induction, while serum induction of c-fos showed a modest MKL-dependence at the later time points of serum induction and was largely independent of MKL at the early time points of induction (30 minutes) [21]. Consistent with this, Gineitis et al. identified c-fos as a gene whose serum induction is largely dependent on the MAPK-TCF pathway [19]. Overexpression of MKL1 has different effects on specific SRF target gene promoters, as some were strongly activated (e.g. smooth muscle α-actin, SM22) while others were poorly induced (e.g. c-fos, egr-1)[21]. The lower activation of the c-fos promoter is not due to the c-fos SRE per se, since reporter genes containing the c-fos SRE on a minimal promoter were strongly activated by MKL1 [21,27].

Chromatin immunoprecipitation experiments have shown that MKL1 binds to the promoters of the cyr61, srf and vinculin genes but not egr-1 or c-fos after swinholide treatment which leads to the activation of the Rho-actin pathway [23]. Moreover SAP-1 (a TCF factor) bound to the egr-1 promoter but not to the srf, vinculin or cyr61 promoters suggesting that TCF and MKL1 binding to target promoters might be mutually exclusive. Indeed, in vitro gel mobility shift assays suggest that MKL1 and Elk-1 bind to the same region of SRF [23]. Nevertheless, it is still unclear how MKL family factors distinguish SRF target genes. It is possible that there are specific CArG box sequences, that TCF sites adjacent to SREs inhibit activation or that other flanking elements control sensitivity to MKL activation.

As a first step towards a better understanding of this differential target gene selection and to demonstrate the importance of the MKL coactivators in immediate early gene induction, we sought to identify the group of SRF target genes that were dependent on the RhoA-MKL pathway. We performed microarray analysis using cell lines expressing dominant negative MKL1 (DN-MKL1) to identify MKL-dependent and -independent serum-induced SRF target genes. The DN-MKL1 protein is effective in blocking both MKL1 and 2 activation of SREs, thus solving the problem of redundancy of these proteins in the cell [21,24](unpublished data). As a control, DN-MKL1 did not affect TCF-pathway induction of reporter genes [21]. Hence, cell lines expressing DN-MKL1 can be used to elucidate the target genes of SRF that require MKL1/2 for their activation. We have further searched the target promoters for common regulatory elements, in particular for perfect or variant CArG box sequences to identify promoters that are more likely to be direct targets of SRF-MKL activity.

## Results

### Dominant negative MKL1 inhibits serum induction of SRF target genes

**Figure 1:**
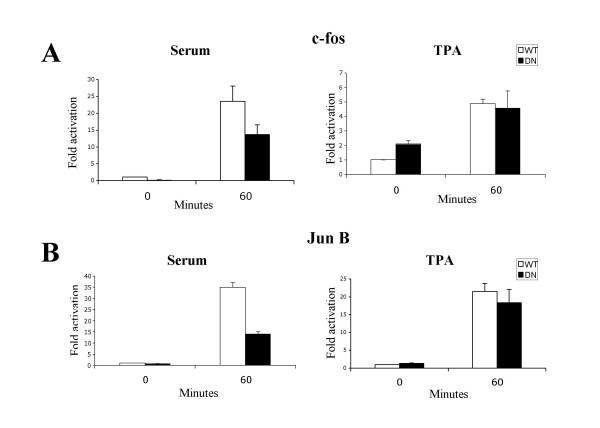
**Effect of serum and TPA on gene expression in WT and DN-MKL1 cell lines. **Serum starved cells were treated with new born calf serum (20%) or TPA (100 ng/ml) for 1 hour and relative mRNA levels for c-fos and jun B were measured by quantitative real-time PCR using the SYBR green method. Data are represented as the relative fold activation ± the standard deviation of the induced cells compared to the serum starved WT cells.

We have utilized a C-terminal deletion mutant of MKL1 (a.a. 1-630) as a dominant negative mutant. We previously found that this mutant can prevent activation of SRE reporter genes by MKL1 and MKL2 overexpression [21](unpublished data). This DN-MKL1 mutant can bind to SRF but lacks a transcriptional activation domain. Expression of DN-MKL1 reduced serum induction of a c-fos reporter gene that lacks a TCF site and strongly blocked RhoA activation of this reporter [21]. We generated a cell line stably expressing DN-MKL1 in NIH3T3 cells in order to more easily look at its effect on endogenous gene expression. We found a strong effect on serum-induced expression of the known SRF target genes vinculin and the SRF gene itself [21]. However, binding of dominant negative MKL1 to SRF could titrate other SRF complexing proteins such as the TCF factors Elk1 and SAP-1/2, since they all bind to the MADS box DNA binding domain of SRF [23]. Thus there is a possibility that DN-MKL1 could block the serum induced expression of target genes by indirectly blocking the TCF pathway. We previously found that dominant negative MKL1 does not significantly inhibit tetradecanoyl phorbol acetate (TPA) activation of SRE reporter genes, which functions through MAPK phosphorylation of TCF [12,21]. We sought to further confirm this for endogenous gene targets. Serum starved cells were induced with serum or TPA for 60 minutes and the mRNA from these cells were assayed for the expression of the endogenous target genes c-fos and junB by real-time PCR (Fig. 1). The DN-MKL1 cell line showed a modest decrease in serum induced expression of c-fos, consistent with our previous RNase protection measurements [21]. A larger decrease was observed for serum induction of junB, however there was no significant change in TPA induced expression of either c-fos or c-jun in the DN-MKL1 cell line (Fig. 1). Hence, DN-MKL1 does not block the TCF pathway and effectively distinguishes the MKL and TCF pathways.

### Microarray analysis identifies MKL dependent genes with varying kinetics of expression

In order to more fully characterize the temporal program of serum induced gene expression and the role of MKL, we performed microarray analysis on wt and DN-MKL1 NIH3T3 cells that had been serum-starved and induced with serum for 0, 30, 60 or 120 minutes. These times should distinguish early vs. 'delayed' immediate early genes. Mouse Affymetrix oligonucleotide arrays (MOE430A) containing 14,824 non-redundant probes were used for hybridization. All data points were done in triplicate and hierarchical clustering was performed using dChip software analysis. We found 229 genes that showed a significant variation across the 8 samples and used the results with these genes for unsupervised clustering [30].

**Figure 2:**
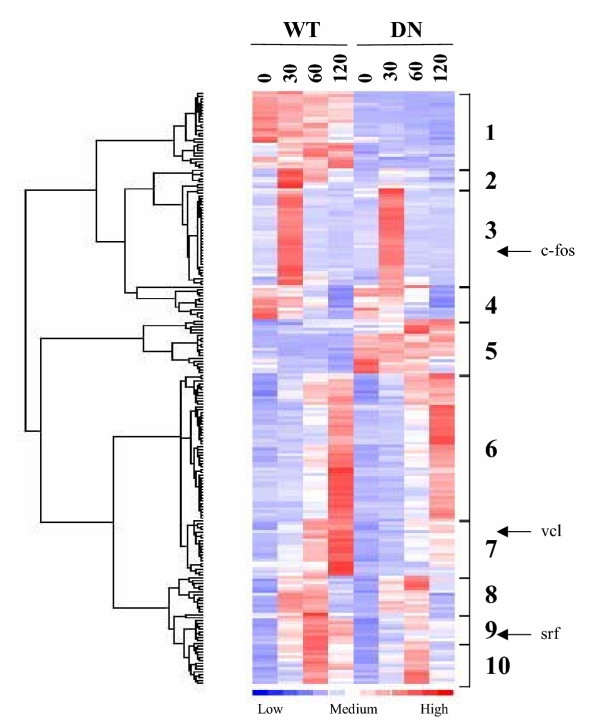
**Hierarchical clustering of gene expression. **RNA from WT and DN-MKL1 expressing cells induced with serum for the indicated times was used to probe an Affymetrix mouse chip with 14,824 non-redundant probes. dChip software was used to identify genes with significant variation across samples. 229 such genes were used for clustering analysis. Different classes of similarly regulated genes are indicated to the right and discussed in the text. The respective positions where the previously characterized MKL target genes srf, vinculin and c-fos fall are indicated by the arrows. Expression scales ranging from -3 (blue) to +3 (red) fold are indicated at the bottom of the figure.

The hierarchical clustering analysis shows classes of differentially regulated genes which we have designated classes 1 to 10 (Fig. 2). We were particularly interested in serum-induced genes. However, one class was constitutively repressed in DN-MKL1 cells (#1) and another was constitutively activated (#5). Further, one class was repressed by serum induction (#4) though there was no significant effect of DN-MKL1. This leaves seven classes of genes that are serum-induced with different kinetics and dependency upon MKL1. Classes 2, 3 and 8 were induced early at the 30 minute time point while classes 6 and 7 had maximal induction at 120 minutes. Classes 9 and 10 had peak induction at the intermediate time point of 60 minutes. Serum induced expression of classes 2, 7 and 9 as well as many of the genes in class 8 were particularly MKL-dependent while the other serum-induced genes (classes 3, 6 and 10) were predominantly MKL-independent. The positions of known SRF target genes that we previously studied are indicated (Fig. 2). The vinculin and srf genes fall into the MKL-dependent classes 7 and 9 consistent with our previous results [21]. Serum-induced expression of the c-fos gene was predominantly independent of MKL1.

To analyze the serum-inducible genes more carefully, we further dissected the data using more stringent criteria to identify MKL-dependent genes at each time point. We filtered for genes that were serum-induced ≥ 2 fold in WT cells and whose expression in DN-MKL1 cells was at least 35% less than in WT cells (Tables 1, 2 and 3). We picked points that fell within the 90% confidence intervals for both fold serum induction and decrease in DN-MKL1 cells. At the 30 minute time point, 12 out of the 52 serum-inducible genes were MKL dependent. Similarly, 6 out of 59 and 18 out of 123 genes were MKL dependent at the 60 and 120 minute time points, respectively. The genes previously shown to contain SRF binding sites are indicated. Consolidating these results to eliminate multiple listings of genes induced at different time points, we have identified 28 out of 150 serum-inducible genes as MKL-dependent (Table 4). A list of the 122 serum inducible, MKL-independent genes is shown in the supplemental table.

**Table 1:** List of genes induced by serum at 30 minutes in WT cells and reduced in DN-MKL1 cells.

	**Gene**	**Locus Link**	**Fold change**	**Lower CI**	**Upper CI**	**% decrease in DN**	**known SRE**
1	similar to Hs Mitogen inducible gene 6	74155	**18.67**	12.81	26.3	**54**	
2	coagulation factor III	14066	**8.05**	5.44	14.29	**47**	
3	retinoblastoma inhibiting gene 1	19649	**6.45**	4.97	8.90	**63**	
4	tropomyosin 1, alpha	22003	**5.92**	4.18	8.55	**63**	**YES**
5	pleckstrin homology-like domain	21664	**5.82**	3.67	11.48	**65**	
6	epiregulin	13874	**5.77**	3.15	9.27	**71**	
7	leukemia inhibitory factor	16878	**4.84**	2.79	7.65	**57**	
8	serum response factor	20807	**4.7**	3.25	6.40	**60**	**YES**
9	tribbles homolog 1	211770	**3.87**	2.81	5.10	**50**	
10	aortic alpha actin-2	68377	**3.77**	2.94	5.06	**52**	**YES**
11	fos-like antigen 1	14283	**3.02**	2.27	3.89	**44**	**YES**
12	CDC42 effector protein 3	260409	**2.47**	2.19	2.81	**46**	

**Table 2:** List of genes induced by serum at 60 minutes in WT cells and reduced in DN-MKL1 cells.

	**Gene**	**Locus Link**	**Fold change**	**Lower CI**	**Upper CI**	**% decrease in DN**	**known SRE**
1	serum response factor	20807	**6.26**	4.66	8.20	**59**	**YES**
2	enigma homolog	56376	**4.75**	3.63	6.28	**61**	
3	adrenomedullin	11535	**4.15**	3.28	5.17	**57**	
4	retinoblastoma inhibiting gene 1	19649	**3.92**	2.68	5.75	**50**	
5	CDC42 effector protein 3	260409	**3.18**	2.40	4.38	**48**	
6	adenosine A2b receptor	11541	**2.56**	2.00	3.24	**40**	

**Table 3:** List of genes induced by serum at 120 minutes in WT cells and reduced in DN-MKL1 cells.

	**Gene**	**Locus Link**	**Fold change**	**Lower CI**	**Upper CI**	**% decrease in DN**	**known SRE**
1	leukemia inhibitory factor	16878	**32.79**	16.31	54.48	**82**	
2	epiregulin	13874	**21.48**	13.61	32.57	**54**	
3	interleukin 6	16193	**7.84**	4.11	12.98	**90**	
4	inhibitor of DNA binding 3	15903	**7.34**	5.86	9.45	**51**	
5	snail homolog 1	20613	**6.14**	4.17	9.00	**55**	
6	serum response factor	20807	**5.83**	4.39	7.61	**53**	**YES**
7	hexokinase 2	15277	**5.43**	3.52	7.51	**52**	
8	TGFB inducible early growth response	21847	**5.39**	3.88	7.42	**53**	
9	enigma homolog	56376	**5.37**	4.26	6.93	**57**	
10	Jun-B oncogene	16477	**5.25**	3.12	7.93	**61**	**YES**
11	vinculin	22330	**4.54**	3.32	6.10	**52**	**YES**
12	expressed sequence AA939927	99526	**4.38**	3.35	5.79	**76**	
13	B-cell translocation gene 2, anti-proliferative	12227	**3.58**	2.34	6.09	**63**	
14	adrenomedullin	11535	**3.30**	2.79	3.91	**47**	
15	methionine adenosyltransferase II, alpha	232087	**3.28**	2.70	3.95	**43**	
16	ELL-related RNA polymerase II	192657	**3.15**	2.34	4.56	**39**	
17	zyxin	22793	**2.70**	2.09	3.37	**46**	
18	transmembrane 4 superfamily member 10 homolog	109160	**2.32**	2.11	2.56	**39**	

**Table 4:** List of genes induced by serum at 30, 60 or 120 minutes whose induction is MKL-dependent or -independent.

	**MKL-Dependent genes (28)**				
	**Known SRF target Genes**		**Other Genes**		**Other Genes**

1	Jun-B	6	adrenomedullin	18	Adenosine A2b receptor
2	serum response factor	7	B-cell translocation gene 2	19	Coagulation factor III
3	fos-like antigen 1	8	CDC42 effector protein 3	20	Leukemia Inhibitory factor
4	tropomyosin 1, alpha	9	enigma homolog	21	Retinoblastoma inhibiting gene 1
5	vinculin	10	epiregulin	22	TGFβ inducible early growth response
		11	hexokinase 2	23	Tribbles Homolog 1
		12	inhibitor of DNA binding 3	24	Aortic alpha actin-2
		13	interleukin 6	25	expressed sequence AA939927
		14	pleckstrin homology-like domain, family A	26	transmembrane 4 superfamily member 10 homolog
		15	similar to Hs Mig6	27	methionine adenosyltransferase II, alpha
		16	snail homolog 1	28	ELL-related RNA polymerase II, elongation factor
		17	zyxin		

	**MKL-Independent genes (122)**				
	**Known SRF target genes**				

1	cysteine rich protein 61 (**Cyr61**)				
2	thrombospondin 1				
3	FBJ osteosarcoma oncogene (**c-fos**)				
4	FBJ osteosarcoma oncogene B (**FosB**)				
5	cysteine rich protein 1 (**Crp1**)				
6	prostaglandin-endoperoxide synthase 2				
7	early growth response 1 (**Egr1**)				
8	early growth response 2 (**Egr2**)				

### Correlation of microarray and real time PCR data

**Figure 3:**
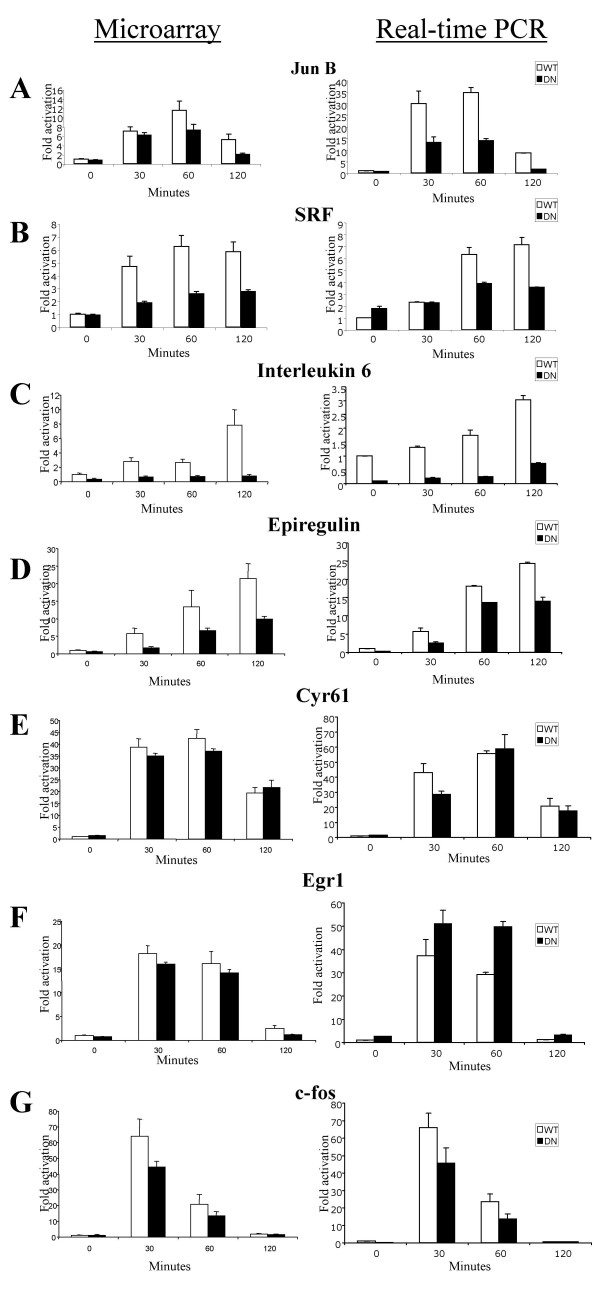
**Correlation of microarray and real-time PCR data**. The expression pattern of select serum-induced MKL-dependent and -independent genes was determined by quantitative real-time PCR (right) and compared to the microarray results (left) for the indicated genes. WT or DN-MKL1 cells were induced with serum for the indicated times before isolation of RNA. The results derived from the microarray hybridizations are the averages of triplicates while the real-time PCR measurements are the averages of at least duplicates ± the standard deviation.

**Figure 4:**
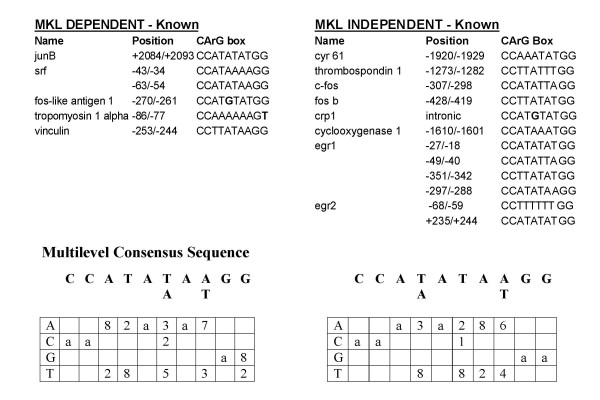
**Known CArG boxes in serum inducible genes. **The upper panel lists the positions and sequences of the known CArG boxes of the MKL-dependent or -independent genes. The bases that differ from the CArG box consensus sequence are in bold. The bottom panel shows the multilevel consensus sequence that was derived from each of these groups of CArG boxes. Below the consensus sequence is the simplified position-specific probability matrix that specifies the probability of each possible base appearing at each position in an occurrence of the motif multiplied by 10. 'a' denotes a probability that is almost or equal to 1. The consensus sequence is the best match to the CArG boxes oriented on either strand.

To verify the results obtained from the microarray experiments, we checked seven genes by real time quantitative PCR using the SYBR Green method. There was a good, but not perfect correlation, of these methods (Fig. 3). Four MKL-dependent genes, jun B, srf, interleukin 6 and epiregulin, were also found to be MKL-dependent by real time PCR. Similarly, two MKL-independent genes, cyr61 and egr-1, were also MKL-independent by real time PCR. Finally, c-fos expression was only moderately reduced in DN-MKL1 cells whether measured by microarray or real time PCR (Fig. 3G). This inhibition was not high enough to meet the cut-off of 35% reduction for our list of MKL-dependent genes (Fig. 4). This result contrasts somewhat with our measurements of c-fos mRNA by RNase protection. We previously found that c-fos mRNA was significantly reduced in DN-MKL1 cells at the 60 minute time point, though not the 30 minute point. Since there was inhibition noted by each method, this difference may reflect the sensitivity of each method to detect precise changes in expression. There were also some differences between the microarray and real time PCR results such as inhibition of Jun B expression at the 30 minute point by the PCR method but not by microarray (Fig. 3A) and the greater serum induction of egr-1 mRNA measured by real time PCR (Fig. 3F). Nevertheless, the general levels of induction and DN-MKL1 inhibition were similar by both methods. This is reflected in the Pearson's correlation coefficient of 0.92 for a comparison of all of the data attained by each method shown in Fig. 3. The correlation coefficient ranged for 0.85 to 0.99 for the data with any one gene.

### Promoter analysis of MKL-dependent and -independent serum-inducible genes

We compared the serum inducible genes to known SRF target genes to identify those genes with SRF binding sites. Comparing to a list of SRF targets genes [31](J.M. Miano, personal communication) we found that five of 28 MKL-dependent genes had known CArG box sites while eight of 122 MKL-independent genes had known sites (Table 4). We further analyzed the promoters of the MKL-dependent genes to identify SRF binding sites that would potentially be direct targets of MKL. Promoter sequences for the serum inducible MKL-dependent or -independent genes were extracted from the Database of Transcription Start Sites (DBTSS) [32]. This database contains exact information of the genomic positions of the transcriptional start sites (based on full length cDNAs) and the adjacent promoters for 6,875 mouse genes. The upstream sequence (-1000 to +200 relative to the transcription start site) of each of the candidate genes was searched for the consensus SRF binding site (CCWWWWWWGG, where W is A or T) with or without one base mismatch allowed. Of the 28 MKL-dependent genes, upstream sequences were available for 20 genes in the DBTSS database while sequence was extracted for 75 of the 122 MKL-independent genes. There were 17 exact matches to the CArG box in these 95 serum inducible promoters (18%) (Table 5A). Given multiple matches in some genes this resulted in 11 genes (12%) having exact matches. These frequencies were similar in the MKL-independent and -dependent classes of serum-inducible genes but significantly higher than the frequencies found in searching the entire DBTSS collection of promoters as indicated by the p values in Table 5A. The p value for the MKL-dependent promoters is high because of the small sample size, however the frequency of exact CArG box matches is similar to the other serum-inducible genes.

**Table 5:** Search of MKL-dependent and -independent serum-inducible promoters for SRF binding sites.

**A. Exact matches to CArG box**											
	genes in micro-array	genes in DBTSS	matches	frequency	p	genes with matches	frequency	p	genes w. >1 matches	frequency	p

Total genes	14824	6875	419	0.061		401	0.058		15	0.002	
All Serum-inducible	150	95	17	0.179	0.0001	11	0.116	0.0218	3	0.032	0.0010
MKL-indep, serum-inducible	122	75	14	0.187	0.0002	9	0.120	0.0293	2	0.027	0.0101
MKL-dep, serum-inducible	28	20	3	0.150	0.1193	2	0.100	0.3245	1	0.050	0.0392

**B. Matches to CArG box allowing one base mismatch**											

Total genes	14824	6875	6707	0.98		4230	0.615		1744	0.254	
All Serum-inducible	150	95	103	1.08	0.155	63	0.663	0.1958	25	0.263	0.4576
MKL-indep, serum-inducible	122	75	72	0.96	0.839	47	0.627	0.4682	15	0.200	0.8886
MKL-dep, serum-inducible	28	20	31	1.55	0.005	16	0.800	0.0666	10	0.500	0.0155

The promoters from indicated groups were searched for exact matches with the CArG box sequence CC(A/T)_6_GG (A) or allowing for one base mismatch (B). Frequency indicates the number of matches divided by the number of DBTSS genes in each category. The p values were calculated based on a binomial distribution except for the value for matches in (B) (column 6 from the left) where the numbers were too large and the Poisson distribution of the frequency of matches per base was used.

We also searched for promoters with more than one CArG box match since multiple SREs have been reported in several SRF target genes and this could provide a mechanism to distinguish responses. While there were more genes with greater than one CArG box match in the serum-inducible genes than in the DBTSS promoters, the numbers of MKL-independent vs. -dependent genes with greater than one match were too low to show a statistical significance (Table 5A).

The search for SRF binding sites allowing a one base mismatch was more problematic since there were a high rate of matches in the DBTSS data set, an average of .98 hits per gene (Table 5B). There was not a significant increase in this rate in the MKL-independent promoters, however there was an increase in the MKL-dependent promoters to 1.55 hits per gene which was found to be statistically significant (p = 0.005; Table 5B). This difference in the MKL-dependent set was also apparent when considering the increased frequency of genes with matches and of genes with greater than one match (Table 5B).

The increased frequency of the exact CArG box sequence in the upstream region of many of the MKL-dependent genes gives further confidence that SRF coordinately regulates many immediate early genes. However, the absence of a CArG box does not preclude a gene from being a direct target due to the flexibility of enhancer positioning. The regulatory site may be upstream or downstream of the 1200 bp we have analyzed. For instance, in the case of the jun B gene it has been shown that a CArG box downstream of the gene mediates its response to serum [33]. In addition not all SRF target sites are perfect CArG boxes. Several of the known SRF target sites contain single base changes (Fig. 4). While our search allowing a single mismatch resulted in an overly high frequency of matches, there was a significantly higher rate of matches in the MKL-dependent promoters suggesting that many of these sites may be used as direct SRF targets.

We compared the CArG boxes more closely since previous work with altered SREs suggested that changes in the A/T core of the SRF can affect sensitivity to the TCF-independent (i.e. RhoA-MKL) pathway [34]. The known CArG boxes were aligned from each class of genes, either MKL-dependent or -independent (Fig. 4). We chose not to analyze the predicted CArG boxes since there were few exact matches in the MKL-dependent promoters and because there was too high a rate of matches allowing one mismatch such that many of the matches are likely false positives. The MEME motif discovery tool (see Methods) was used to arrive at a multilevel consensus sequence based on a position specific probability matrix. The matrix specifies the probability of each possible letter appearing at each position in an occurrence of the motif. MEME also takes into account both orientations of the sequence to arrive at the consensus sequence. The consensus sequences for the two sets are quite similar although there are some minor differences and the sample size is relatively small.

We have compared the sequences of the known SRF target genes to determine whether flanking sequences can explain the differential sensitivity to DN-MKL1. We first compared sequence flanking the known CArG boxes. The MEME motif discovery tool was used to identify common elements in 30 bp flanking each side of the CArG boxes. There were no statistically significant matches in the sequences from MKL-dependent of -independent genes. We might have expected to see a TCF site, however this site is very short and flexible. TCF binds to a short site near an SRE and its binding is stabilized by binding to SRF. A consensus of (C/A)(C/A)GGA(A/T) was found, however the orientation and position relative to the SRE was flexible and only the GGA sequence was absolutely required [35]. Visual inspection of the flanking sequence to the known CArG boxes suggested a potential TCF site in all of the sequences except for that of cyr61.

We used two methods to compare the full promoter sequences of the MKL-dependent and -independent, serum-inducible genes for common elements that might be required for their induction. We first used the MEME tool to compare the promoters (-1000 to +200) derived from the DBTSS database. As in Table 4, 20 MKL-dependent promoter sequences and 75 MKL-independent promoter sequences were extracted. The MEME tool could only compare 50 of these promoters at a time such that 50 MKL-independent promoters and 50 random promoters were compared. While MEME identified some common elements within each of these three classes of promoters, none of the elements identified among the MKL-dependent of -independent promoters appeared to be specific since similar elements were identified in the random promoter set.

A second method we used was to look for common regulatory elements was oligonucleotide analysis which has been used in yeast to identify regulatory sites [36]. This method looks for enriched oligonucleotide frequencies in a group of genes. We used the entire set of 6875 DBTSS mouse promoter sequences to calculate the expected background frequencies of hexamers. The most specific matches we found were in the set of 75 MKL-independent promoters. The related oligonucleotides GGAGGG, CCGGAG and CGGAGA were enriched with significance values (E-value) of 1.4 × 10^-4 ^to 6.9 × 10^-3^. These oligonucleotides were not enriched in a test case of 60 random promoter sequences. The related sequence GGGAGG, however, was similarly enriched in the MKL-dependent promoters albeit with less statistical significance (E = 0.8). It is intriguing that these oligonucleotides are similar to the TCF consensus binding site (C/A)(C/A)GGA(A/T). The most significantly enriched oligonucleotide in the 20 MKL-dependent promoters was CCGCGC with an E value of 0.078. This lower significance may partially reflect the smaller size of this data set but also may reflect the lower significance of this enrichment. Given the relatively small number of serum inducible genes and present computational tools, it appears that more careful experimental mapping of the sequence elements in MKL-dependent and -independent genes will be required to identify the elements that determine their sensitivity to the MKL pathway.

## Discussion

Serum inducible genes have far ranging effects in many physiological processes such as proliferation, wound healing, migration, and tissue remodeling [37]. SRF has been implicated in the expression of many serum inducible genes, particularly immediate early transcription factors, but is also required for the expression of many muscle-specific genes [31]. SRF is activated by different co-activators to control its diverse set of target genes in different tissues [27,38-45]. Here, we have elucidated the target genes that are dependent on one particular SRF co-activator family – the MKL family. Since dominant negative MKL1 can block activation by all of the members of the MKL family, MKL1 and 2, as well as myocardin (unpublished data)[21,24], we have used cell lines expressing dominant negative MKL1 to determine the role of the MKL family in serum-induced gene expression patterns.

Cluster analysis of the microarray data identified different classes of genes that showed significant variation across the samples. Two classes of genes were either constitutively activated or constitutively repressed. These could be indirect targets of the SRF-MKL pathway or could suggest functions for MKL apart from its role as a transcriptional co-activator of SRF.

Among the serum inducible genes, we identified a significant fraction, 28 of 150, that are MKL-dependent. This is not to say that the other 122 genes may not be sensitive to MKL under certain conditions. Rather, they must at least have additional mechanisms for serum induction in NIH3T3 cells. c-fos was identified as an MKL-independent gene although we previously found by RNase protection that its induction was significantly reduced in cells induced with serum for 60 minutes [24]. We did observe a small decrease in c-fos expression in our microarray experiments, consistent with the RNase protection results, however it did not pass our stringent statistical criteria for MKL-dependence. Serum induction of some of the MKL-independent immediate early genes may also be independent of SRF. For example, the c-jun gene utilizes other sequence elements for serum induction [46]. The TCF factors, elk-1, SAP1 and SAP2, are the main candidates besides MKL factors for mediating serum induction of SRF target genes. Further expression profiling will be required with inhibitors of the SRF and TCF pathways to better characterize the pathways used by the 150 serum inducible genes described here.

Many of the serum-inducible genes identified here have been previously described as immediate early genes either as single genes or in microarray experiments [37,47,48]. The groups of genes include immediate early transcription factors and cytokines, as previously noted [37], but also a broad array of other types of genes. SRF null mice die in utero due to the defective formation of a mesodermal layer and SRF null ES cells are defective in focal adhesion and migration [49,50]. We have also observed that the DN-MKL1 cell line is significantly less adherent than the control line (unpublished data). It is therefore interesting that a number of our serum inducible genes are involved in cytoskeletal structure and adhesion. These include vinculin, tropomyosin, zyxin, thrombospondin, tenascin, integrin α5, and transgelin. It will be interesting to determine whether lowered expression of these genes in SRF or MKL deficient cells leads to the changes in their adhesive properties. Of these proteins, only vinculin is a previously known MKL and SRF target gene [19,21,51]. Our microarray analysis found that zyxin is also an MKL-dependent gene and zyxin expression was previously found to be decreased in SRF null cells [50].

### Sequences required for MKL-dependent and -independent genes

We analyzed the promoter sequences of the MKL-dependent and -independent genes to identify elements that might determine this sensitivity. Exact matches to a simple CArG box consensus, CC(A/T)_6_GG, resulted in matches in only about 12% of the serum-inducible genes with similar proportions in the MKL-dependent and -independent classes. While some functional SRF binding sites contain a single base mismatch to the consensus, a broader search for CArG boxes allowing a mismatch resulted in a high number of matches in a broad promoter database. There was a modest, statistically significant increase in the matches in the MKL-dependent promoters suggesting that some of these sites are real SRF targets. Other SRF target sites may lie outside of the promoter regions we have searched (-1000 to +200). This still leaves the possibility that induction of many of the immediate early genes is independent of SRF such as we have found for the c-jun gene [46]. Microarray analysis of serum-inducible genes with inhibition of SRF activity is required to show which genes are SRF dependent.

Possible mechanisms for determining sensitivity to MKL are the sequence of the CArG box, flanking sequence or the context of other promoter elements. Our comparison of the known CArG boxes from MKL-dependent and -independent promoters did not show a strong difference. Similarly we were not able to identify differences in the flanking sequence. Analysis of the more full promoter sequences has also not yielded clear common regulatory elements to date. One set of oligonucleotides, containing the sequence GGAG, was found in both the MKL-dependent and -independent promoters at a significantly higher frequency than in the full promoter database. The significance of these sites remains to be determined though it is notable that they are similar to the TCF consensus site.

One possible mechanism for distinguishing MKL target genes is the presence of a TCF site next to a CArG box. This would allow for TCF binding and activation by a MAP kinase pathway instead of the MKL pathway. Since the binding sites for TCF and MKL on SRF are similar [23], TCF binding could preclude MKL activation. In fact, mutation of the TCF site in the c-fos promoter allows it to be activated by a serum induced, actin filament-dependent pathway presumably through RhoA and MKL [20]. In addition, we found that mutation of the TCF site in the c-fos SRE results in significantly higher activation by MKL1 (Bo Cen and R.P., unpublished results). Nevertheless, c-fos SRE elements with TCF sites are still activated by MKL1 and myocardin [21,27] such that there may be additional elements that determine sensitivity to MKL1 activation.

Chromatin immunoprecipitation experiments have shown that MKL1 can bind to the SRF, vinculin and cyr61 promoters in cells treated with the actin inhibitor swinholide A [23]. This is consistent with our identification of SRF and vinculin as MKL target genes, but we found that induction of cyr61 was MKL-independent. It is possible that this difference is due to our inducing with serum rather than swinholide A, since serum will induce alternative pathways such as the MAP kinase pathway. Chromatin immunprecipitations showed that the TCF factor SAP1 bound to the egr1 promoter, possibly explaining its independence of MKL, but no binding of SAP1 was observed for two other MKL1-independent genes egr-1 and cyr61 [23]. Thus, TCF binding may not always explain the lack of requirement for MKL factors.

## Conclusions

Our results indicate that a subset of serum inducible genes is dependent upon the MKL family for its induction. This genomic classification of MKL-dependent and -independent serum-inducible genes is a significant step for characterizing which pathways are required for induction of each cellular immediate early gene.

## Methods

### Gene expression analysis

NIH3T3 cells stably expressing dominant negative MKL1 (a.a. 1-630)(DN-MKL1) or containing the vector, pBabePuro, were previously described [21]. The cells were serum starved in Dulbecco's modified Eagle's medium (DMEM) with 0.2% new born calf serum (NCS) for 24 hours and then induced with 20% NCS for 30, 60 and 120 minutes. Total RNA from these cells was prepared using Trizol (Invitrogen) and then purified using RNeasy columns (Qiagen). Total RNA (8 μg) was used for first-strand cDNA synthesis using T7-Oligo-dT primers and Powerscript reverse transcriptase (Invitrogen) for 1 hour at 42°C. This was followed by second-strand synthesis for 2 hours at 16°C using RNase H, *E. coli *DNA polymerase I, and *E. coli *DNA ligase (Invitrogen). The obtained double-stranded cDNA was then blunted by the addition of 20 units T4 DNA polymerase and incubation for 5 min at 16°C. The material was then purified by phenol:chloroform:isoamyl alcohol extraction followed by precipitation with ammonium acetate and ethanol. The cDNA was then used in an in vitro transcription reaction for 6 hours at 37°C using a T7 in vitro transcription kit (Affymetrix) and biotin-labeled ribonucleotides. The obtained cRNA was purified on an RNeasy column. The eluted cRNA was then fragmented by incubation of the products for 30 min in fragmentation buffer (40 mM Tris-acetate, pH 8.1, 100 mM KOAc, 30 mM MgOAc) at 95°C. The fragmented labeled RNA (15 μg) was hybridized to an Affymetrix mouse MOE430A Genechip at 45°C according to the manufacturer's protocol [52] and stained with streptavidin-phycoerythrin. The chips were then scanned with an Affymetrix Genechip Scanner GS300. All the data sets were done in triplicates from serum stimulation of cells to scanning of the microarrays.

The percent of genes with significant expression ("present" calls) ranged from 50.4 to 56.5% for each microarray. The 3':5' ratio for a control actin gene ranged from 1.0 to 1.31 for each of the 24 microarrays scanned. The scaling factor to account for differences in probe labeling and general changes in hybridization in each microarray was scaled to a target intensity of 250 (using Affymetrix Microarray Suite 5.0 software) and ranged from 1.273–5.542.

### dChip analysis

The dChip software [30,53] was used for the normalization and calculation of model based expression indexes (MBEI) after pooling the replicate arrays and for the hierarchical clustering analysis of the data from the Affymetrix Gene chips. Normalization was based on a large set of probes determined iteratively to be invariant across the different microarrays. After normalization each array has a similar overall signal [30]. The initial analysis was performed using the perfect match (PM)-only model and genes were filtered according to the criteria of a) co-efficient of variation (standard deviation/mean) across the eight conditions must be between 0.50 and 10.00, b) a gene must be called 'Present' in ≥ 20% of the arrays used, c) the variation of the standard deviation/mean for replicate arrays for a single condition must be between 0 and 0.5, and d) the expression level must be >= 100 in at least one of the time points. Hierarchical tree clusters were then generated using this filtered gene list.

Expression values (MBEIs) at each time point were determined with a standard error of the mean. These values were used to calculate the fold change with 90% confidence intervals. The confidence intervals were derived from the standard errors and fold changes based on a χ^2 ^distribution model with one degree of freedom [30]. For the lists of serum inducible genes the low confidence interval value was required to be greater than two. For the inhibition in the DN-MKL cells, the low confidence interval value was required to be at least 35% less than in the WT cells for the gene to be designated MKL-dependent.

### Real time PCR

Total RNA was isolated from the DN-MKL1 and vector containing NIH3T3 cells as described for the microarray analysis and 1 μg was used for first strand cDNA synthesis (Powerscript Reverse Transcriptase, BD Biosciences) using oligo-dT primers according to the manufacturer's protocols. One fiftieth of the reverse transcription reaction was included in a 20 μl PCR reaction. For a quantitative analysis, SYBR green PCR technology was used (Applied Biosystems). Real-time detection of the PCR product was monitored by measuring the increase in fluorescence caused by the binding of SYBR green to double-stranded DNA with an ABI PRISM 7000 Sequence Detector. To calculate relative quantification values a threshold cycle (C_t_), the cycle at which a statistically significant increase in fluorescence occurs, was derived from the resulting PCR profiles of each sample. C_t _is a measure of the amount of template present in the starting reaction. To correct for different amounts of total cDNA in the starting reaction, C_t _values for an endogenous control (Acidic ribosomal phosphoprotein P0) were subtracted from those of the corresponding sample, resulting in ΔC_t_. The ΔC_t _value of the serum starved wt sample was chosen as the reference point and was subtracted from the ΔC_t _value of all the other samples, resulting in ΔΔC_t_. The relative quantification value is expressed as 2^-ΔΔCt ^giving the relative difference of the serum induced points compared to the serum-starved cells for expression of a particular gene. All real time PCR data sets shown are the results of two independent mRNA preparations and amplifications. Amplification of only a single species in each PCR reaction was determined by checking for a dissociation curve with a single transition.

### Promoter sequence analysis

The Database of Transcriptional Start Sites (DBTSS)[32,54] was used to extract the promoter sequences (-1000 to +200) of all the available full length cDNAs and RefSeq entries based on the start site of the cDNA as +1. This mouse data base was searched for SRF binding sites using the consensus CCWWWWWWGG, where W is A or T, with or without allowing for one base mismatch.

The CArG box sequences of the known SRE regulated genes were derived from the literature. The motif discovery tool MEME (Multiple EM for Motif Elicitation) [55,56] was then used to derive a multilevel consensus sequence based on a position specific probability matrix. The matrix specifies the probability of each possible nucleotide appearing at each possible position in an occurrence of the motif. MEME takes into account both orientations of the sequence to arrive at the consensus sequence. Sequences flanking the known SREs (30 bp on each side) were extracted from Genbank and MEME was also used to compare these sequences. A control set of 10 upstream sequences from the same genes (70 bp in length) was also searched for comparison.

Promoter sequences (-1000 to +200) of the serum inducible genes were also searched for common elements with MEME. Promoters for 20 MKL-dependent, 50 MKL-independent and 50 random promoters were searched. These promoter sequences were additionally searched by oligonucleotide analysis for enriched hexamers [36,57] except that 20 MKL-dependent and 75 MKL-independent were searched. The background frequency of oligonucleotides was set using promoter sequence (-1000 to +200) for all 6875 mouse promoters in DBTSS. A control searching 60 random promoters also did not show significant enrichment of specific oligonucleotides.

## Authors' contributions

AS carried out all experimental sections of the paper. AS and RP conceived of the study and participated in its design and coordination. Both authors read and approved the final manuscript.

## Supplementary Material

Additional File 1 **List of serum inducible MKL-independent genes**This Microsoft Excel spreadsheet file contains a list of genes that were serum inducible (>2-fold) at either the 30, 60 or 120 minute time points and that satisfied the 90% confidence interval criteria for fold-change using the dChip software. The expression of these genes were reduced by less than 35% in the DN-MKL1 cells at each time point. The MKL-dependent genes (reduced by more than 35%) shown in Tables 1 to 3 were removed from this list to give 122 MKL-independent genes. The Affymetrix probe set information, the gene name, Genbank Accession number and Locus link identifier are shown for each of the genes. For each time point the fold induction in serum-treated wt NIH3T3 cells compared to serum-starved wt cells ('fold change') and its upper and lower confidence intervals are shown. The time points where serum induction was deemed significant for each gene are marked by asterisks (*) in the 'filtered' columns. Click here for file
